# Emergence of Omicron FN.1 a descendent of BQ.1.1 in Botswana

**DOI:** 10.1093/ve/veae095

**Published:** 2024-11-22

**Authors:** Wonderful T Choga, Emanuele Gustani-Buss, Houriiyah Tegally, Dorcas Maruapula, Xiaoyu Yu, Monika Moir, Boitumelo J L Zuze, San Emmanuel James, Nokuthula S Ndlovu, Kedumetse Seru, Patience Motshosi, Alexandra Blenkinsop, Irene Gobe, Cheryl Baxter, Justen Manasa, Shahin Lockman, Roger Shapiro, Joseph Makhema, Eduan Wilkinson, Jason T Blackard, Phillipe Lemey, Richard J Lessells, Darren P Martin, Tulio de Oliveira, Simani Gaseitsiwe, Sikhulile Moyo

**Affiliations:** Research Laboratory, Botswana Harvard Health Partnership, Gaborone, Private Bag BO 320, Botswana; Faculty of Health Sciences, School of Allied Health Sciences, Gaborone, Private Bag UB 0022, Botswana; Centre for Epidemic Response and Innovation (CERI), School of Data Science and Computational Thinking, Stellenbosch University, Stellenbosch 7600, South Africa; Laboratory for Clinical and Epidemiological Virology, Department of Microbiology, Immunology and Transplantation, Rega Institute, KU Leuven, Leuven 3000, Belgium; Centre for Epidemic Response and Innovation (CERI), School of Data Science and Computational Thinking, Stellenbosch University, Stellenbosch 7600, South Africa; Research Laboratory, Botswana Harvard Health Partnership, Gaborone, Private Bag BO 320, Botswana; Institute of Evolutionary Biology, University of Edinburgh, Edinburgh EH9 3FL, Scotland, UK; Centre for Epidemic Response and Innovation (CERI), School of Data Science and Computational Thinking, Stellenbosch University, Stellenbosch 7600, South Africa; Research Laboratory, Botswana Harvard Health Partnership, Gaborone, Private Bag BO 320, Botswana; Faculty of Health Sciences, School of Allied Health Sciences, Gaborone, Private Bag UB 0022, Botswana; KwaZulu-Natal Research Innovation and Sequencing Platform (KRISP), School of Laboratory. Medicine and Medical Sciences, University of KwaZulu-Natal, Durban 4001, South Africa; Research Laboratory, Botswana Harvard Health Partnership, Gaborone, Private Bag BO 320, Botswana; Research Laboratory, Botswana Harvard Health Partnership, Gaborone, Private Bag BO 320, Botswana; Research Laboratory, Botswana Harvard Health Partnership, Gaborone, Private Bag BO 320, Botswana; Department of Mathematics, Imperial College London, London, Westminster, SW7 2AZ, United Kingdom; Faculty of Health Sciences, School of Allied Health Sciences, Gaborone, Private Bag UB 0022, Botswana; Centre for Epidemic Response and Innovation (CERI), School of Data Science and Computational Thinking, Stellenbosch University, Stellenbosch 7600, South Africa; Faculty of Medicine and Health Sciences, Molecular Diagnostics and Investigative Sciences, University of Zimbabwe, Harare, P.O.Box MP167, Zimbabwe; Research Laboratory, Botswana Harvard Health Partnership, Gaborone, Private Bag BO 320, Botswana; Department of Immunology and Infectious Diseases, Harvard T.H. Chan School of Public Health, Boston, MA 02115, United States; Division of Infectious Diseases, Brigham & Women’s Hospital, Boston, MA 02115, United States; Harvard Medical School, Boston, MA, 02115, United States; Research Laboratory, Botswana Harvard Health Partnership, Gaborone, Private Bag BO 320, Botswana; Department of Immunology and Infectious Diseases, Harvard T.H. Chan School of Public Health, Boston, MA 02115, United States; Research Laboratory, Botswana Harvard Health Partnership, Gaborone, Private Bag BO 320, Botswana; Department of Immunology and Infectious Diseases, Harvard T.H. Chan School of Public Health, Boston, MA 02115, United States; Centre for Epidemic Response and Innovation (CERI), School of Data Science and Computational Thinking, Stellenbosch University, Stellenbosch 7600, South Africa; University of Cincinnati College of Medicine, Cincinnati, OH 45267, United States; Laboratory for Clinical and Epidemiological Virology, Department of Microbiology, Immunology and Transplantation, Rega Institute, KU Leuven, Leuven 3000, Belgium; KwaZulu-Natal Research Innovation and Sequencing Platform (KRISP), School of Laboratory. Medicine and Medical Sciences, University of KwaZulu-Natal, Durban 4001, South Africa; Division of Computational Biology, Department of Integrative Biomedial Sciences, Institute of Infectious Diseases and Molecular Medicine, University of Cape Town, Cape Town 7925, South Africa; Centre for Epidemic Response and Innovation (CERI), School of Data Science and Computational Thinking, Stellenbosch University, Stellenbosch 7600, South Africa; KwaZulu-Natal Research Innovation and Sequencing Platform (KRISP), School of Laboratory. Medicine and Medical Sciences, University of KwaZulu-Natal, Durban 4001, South Africa; Department of Global Health, University of Washington, Seattle, WA 98105, United States; Research Laboratory, Botswana Harvard Health Partnership, Gaborone, Private Bag BO 320, Botswana; Department of Immunology and Infectious Diseases, Harvard T.H. Chan School of Public Health, Boston, MA 02115, United States; Research Laboratory, Botswana Harvard Health Partnership, Gaborone, Private Bag BO 320, Botswana; Department of Immunology and Infectious Diseases, Harvard T.H. Chan School of Public Health, Boston, MA 02115, United States; School of Health Systems and Public Health, University of Pretoria, Pretoria 0002, South Africa; Division of Medical Virology, Faculty of Medicine and Health Sciences, Stellenbosch University, Tygerberg, Cape Town 7602, South Africa

**Keywords:** Omicron FN.1, phylodynamics, SARS-CoV-2, Botswana, Africa

## Abstract

Botswana, like the rest of the world, has been significantly impacted by severe acute respiratory syndrome coronavirus 2 (SARS-CoV-2). In December 2022, we detected a monophyletic cluster of genomes comprising a sublineage of the Omicron variant of concern (VOC) designated as B.1.1.529.5.3.1.1.1.1.1.1.74.1 (alias FN.1, clade 22E). These genomes were sourced from both epidemiologically linked and unlinked samples collected in three close locations within the district of Greater Gaborone. In this study, we assessed the worldwide prevalence of the FN.1 lineage, evaluated its mutational profile, and conducted a phylogeographic analysis to reveal its global dispersal dynamics. Among approximately 16 million publicly available SARS-CoV-2 sequences generated by 30 September 2023, only 87 were of the FN.1 lineage, including 22 from Botswana, 6 from South Africa, and 59 from the UK. The estimated time to the most recent common ancestor of the 87 FN.1 sequences was 22 October 2022 [95% highest posterior density: 2 September 2022—24 November 2022], with the earliest of the 22 Botswana sequences having been sampled on 7 December 2022. Discrete trait reconstruction of FN.1 identified Botswana as the most probable place of origin. The FN.1 lineage is derived from the BQ.1.1 lineage and carries two missense variants in the spike protein, S:K182E in NTD and S:T478R in RDB. Among the over 90 SARS-CoV-2 lineages circulating in Botswana between September 2020 and July 2023, FN.1 was most closely related to BQ.1.1.74 based on maximum likelihood phylogenetic inference, differing only by the S:K182E mutation found in FN.1. Given the early detection of numerous novel variants from Botswana and its neighbouring countries, our study underscores the necessity of continuous surveillance to monitor the emergence of potential VOCs, integrating molecular and spatial data to identify dissemination patterns enhancing preparedness efforts.

## Introduction

Severe acute respiratory syndrome coronavirus 2 (SARS‐CoV‐2) is a single-stranded, positive-sense RNA virus with a ∼29.9 -kb genome belonging to the *Betacoronavirus* genus of the family *Coronaviridae* (Huang et al. 2020). Its associated disease, COVID-19, has resulted in nearly 7 million deaths since its discovery in 2020 (WHO 2023). Viruses are subject to a variety of evolutionary pressures acting upon mutations that frequently arise as a result of error-prone RNA replication and host-mediated RNA editing systems. Since mutations occur at a rate commensurate with their global spread, the mutational landscape of SARS-CoV-2 can be used to track its geographical dissemination dynamics over time (van Dorp et al. 2020).

As viruses mutate, variants may emerge that exhibit distinct degrees of fitness. The emergence of variants of concern (VOC) lineages with increased fitness (i.e. Alpha, Beta, Gamma, Delta, and Omicron) precipitated successive waves of the global COVID-19 pandemic, during which newly emerged variants displaced preceding variants. Omicron VOC and its sublineages are able to evade responses from natural and vaccine-induced immunity and often display an increased binding affinity for angiotensin-converting enzyme 2 (ACE2) receptors, suggesting adaptive evolution (Huo et al. 2023, Ma et al. 2023). Omicron sublineages are also commonly characterized by high transmission potential and frequently demonstrate distinct global distribution patterns (Tamura et al. 2023). Notably, the proliferation of each VOC led to a resurgence of infections across the globe (Gangavarapu et al. 2023), representing a formidable challenge to sustained pandemic surveillance efforts.

As in some other African countries, Botswana implemented an effective genomic surveillance programme to track the diversification and dispersal dynamics of SARS-CoV-2 variants in near real-time (Choga et al. 2024a, 2024c). The SARS-CoV-2 genomic surveillance in Botswana is an integrated two-phased genomic surveillance system that identifies circulating lineages and examines domestically emerged lineages to determine potential variants under monitoring (VUM), while also tracking the spread of known VUM, variants under investigation (VUI), and VOCs. This surveillance program enabled the early detection of novel variants such as B.1.1.529 (alias BA.1), designated as Omicron by the World Health Organization (WHO) (Viana et al. 2022). Relative to previously characterised VOCs, Omicron has continued to evolve since its emergence. For example, after Omicron BA.5 emerged between mid-December 2021 and early January 2022 (Tegally et al. 2022a), multiple genetically distinct sublineages of BA.5 arose in different parts of the world. Notably, by July 2022, Nigeria had reported the BQ.1 and BQ.1.1 sublineages among the immediate descendants of BA.5 (Akash et al. 2023). BQ.1.1 (alias B.1.1.529.5.3.1.1.1.1.1.1)—descending from BQ.1—had accumulated multiple “lineage-defining” mutations including the R346T, K444T, L452R, N460K, and/or F486V mutations within the spike glycoprotein (Ito et al. 2023), and N1191S in ORF1b. On 12 October 2022, the WHO designated BQ.1 and its sublineages (BQ.1.*) as an Omicron VUM (WHO 2024).

The ongoing, near real-time genomic surveillance program in Botswana with routine sequencing of randomly selected samples from all nine COVID-19 zones of the country, facilitated tracking of the diversification and subsequent emergence of BQ.1*. In early December 2022, we identified FN.1, a new descendant of BQ.1.1. FN.1 is a domestic variant that meets the criteria for further investigation as a typical example of a SARS-CoV-2 lineage that has emerged and spread likely originating from Botswana. It has shown not only local transmission clusters but has also been exported internationally, resulting in a minor outbreak. To this end, we have demonstrated what can be done to detect and evaluate the level of threat of emerging SARS-CoV-2 lineages using our surveillance system. We employed an integrative approach combining genetic and protein structural analyses of FN.1. Additionally, we analyzed the clinical characteristics of FN.1, compared its mutational profile to that of its parental lineage, BQ.1.1, and evaluated its close relationship to lineages circulating in Botswana both during and prior to FN.1’s emergence.

## Methods and materials

### Ethics, study design, and sample collection

Retrospective analysis conducted as part of the SARS-CoV-2 genomic surveillance of routine diagnostic testing samples was approved by the Health Research and Development Committee in Botswana (Protocol# HRDC00945; HPDME 13/18/1), the Harvard T.H Chan School of Public Health Office of Research Administration (Protocol #IRB21-1661), and the Mass General Brigham Institutional Review Board (Protocol#2022P00421). Combined nasopharyngeal and oropharyngeal (N/O) samples routinely collected in various regions of the country were sent to the nearest COVID-19 polymerase chain reaction (PCR) testing laboratory for SARS-COV-2 testing using the criteria previously described (Choga et al. 2024b). SARS-CoV-2 sequencing and analysis were conducted at the Botswana Harvard Health Institute Partnership in Gaborone, which serves as the main referral Center for SARS-CoV-2 genomic surveillance in the country.

### SARS-COV-2 testing using real-time polymerase chain reaction

Nucleic acid extraction was carried out using the MGI Sample Preparation 960 and the Nucleic Acid Extraction Kit (Wuhan MGI Tech Co., Ltd, Wuhan, China) according to the manufacturer’s instructions. Isolated nucleic acid was stored at −70°C until PCR. For diagnostic qPCR analyses, we used the 2019-nCoV RNA (PCR-Fluorescence Probing) Assay (Sun Yat-sen University, Da An Gene Co., Ltd, China) according to the manufacturer’s instructions that target the nucleocapsid (N) and Open reading Frame 1ab (ORF1ab). An extraction control (RNase free water), master-mix-only control, positive, and non-template controls were included in each PCR.

### SARS-CoV-2 whole-genome amplification using tiling PCR

The midnight protocol for Oxford Nanopore Technology (ONT) sequencing was utilized as described previously (Freed et al. 2020). Gene-specific multiplex PCR with midnight primer pools generated 1.2-kb amplicons covering the entire SARS-CoV-2 genome. Amplicons from each pool were combined and barcoded using the ONT Rapid Barcoding kit (Oxford Nanopore Technologies, Oxford, UK), and the library was loaded onto a prepared R9.4.1 flow-cell.

### Nanopore sequencing and raw data processing

GridION machines (ONT) were used for sequencing, and the latest MinKNOW release version (depending on the time of analysis) was set for demultiplexing and base calling with either fast or high accuracy mode. An ad hoc script was used to concatenate all reads (raw FASTQ) produced per each barcode. Each concatenated FASTQ file was analyzed using Genome Detective (GD) (Vilsker et al. 2019). Binary Alignment Map (BAM) and consensus FASTA files were obtained as the output of GD.

### Quality control and lineage assignment of SARS-CoV-2

To assess the quality control reports, NextClade version 2.14.1 (Aksamentov et al. 2021) was used and the alignment was manually visualized and polished in AliView (Larsson 2014), as previously described (Viana et al. 2022, Tegally et al. 2022a). A dynamic lineage classification method from the Phylogenetic Assignment of Named Global Outbreak LINeages tool (PANGOLIN) software suite (Rambaut et al. 2020, 2021) was used to designate the sequences to known SARS-CoV-2 lineages.^8,19^ The final consensus edited sequences and associated metadata were deposited in the Global Initiative on Sharing all Influenza Data (GISAID) database (Khare et al. 2021).

### Maximum-likelihood phylogenetic analysis

All sequences were aligned using NextAlign (Aksamentov et al. 2021) to obtain a codon alignment of the sequences. A maximum-likelihood (ML) tree was inferred from the resulting alignment in IQ-TREE 2 (Minh et al. 2020) using the TN model with empirical base frequencies with gamma distributed over site-to-site variations in nucleotide substitution rates, as determined by jModelTest2 (Darriba et al. 2012). Statistical supports for nodes of the ML phylogeny were assessed using a bootstrap approach with 1000 replicates. The reliability of the observed clades was established based on internal node bootstrap values exceeding 80%.

### Molecular clock signal estimation

The topology of the ML tree was inspected in TempEst ver.1.5.3 (Rambaut et al. 2016) for the presence of a temporal signal. TempEst (Rambaut et al. 2016) plots linear regression of sampling dates against root-to-tip genetic distances obtained from the ML phylogeny. The plot showed that the FN.1 sequences evolved in a relatively strong clock-like manner with sufficient temporal signal for molecular clock analysis (correlation coefficient = 0.67 and *R*^2^ = 0.45).

### Phylodynamic analysis and visualizations

To estimate time-calibrated phylogenies dated using time-stamped genome data, Markov Chain Monte Carlo (MCMC) methods within the BEAST software ver.1.10.4 were also employed to conduct Bayesian time-scaled phylogenetic analyses (Suchard et al. 2018). We used two independent runs of 50 and 100 million MCMC steps with exponential and SkyGrid coalescent priors with model parameter values and trees sampled every 5000 steps and 10 000 steps, respectively (Drummond et al. 2005). Ancestral reconstruction of discrete ancestral geographical location states in the Bayesian framework was implemented with the SkyGrid coalescent run, using the Bayesian asymmetric discrete trait evolution model (Suchard et al. 2018). A Bayesian stochastic search variable selection (BSSVS) approach was used in order to find a minimal set of rates explaining the diffusions in the phylogeny. Rates yielding a BF of > 3 were considered significant. In each run, a SRD06 substitution model (Shapiro et al. 2006) was used, which employs a Hasegawa, Kishino, and Yano (HKY) nucleotide substitution model, gamma distributed site-to-site mutation rate variation (HKY + Γ) (Shapiro et al. 2006). The evolutionary rates were estimated under a strict clock rate model (Drummond et al. 2006). Demographic estimations of both coalescent models were implemented to gauge the median effective population size (*N_e_*) over time, accompanied by a 95% highest posterior density (95% HPD) interval. The SkyGrid and exponential effective population estimates were plotted using Vega lite (Satyanarayan et al. 2017, Zong et al. 2023). Convergence was evaluated using Tracer ver.1.7.2 with parameters having an ESS value of over 200 (Suchard et al. 2018), with removal of the 10% sampled states to accounted for burn-in. TreeAnnotator ver.1.10.4 was employed to generate maximum clade credibility (MCC) trees.

Annotation and visualization of the MCC phylogeny were done using the *ggtree* (Yu et al. 2016) package of the R software platform (http://www.R-project.org/) and the different coalescent model tree comparisons were displayed in Auspice ver.2.36 (Hadfield et al. 2018). The reliability of the observed clades was established based on posterior probability values with significance levels of ≥ 0.8. TMRCA estimates were expressed as the median and 95% HPD intervals of years prior to the oldest sampling date, which corresponded to the first samples (2022) reported in this study. This study did not employ a predetermined sample size using statistical methods. Geographical information system (GIS)-based maps showing the distribution of FN.1 were produced using the open-source QGIS software (https://qgis.org).

### Antigenicity, pathogenicity, and immunogenicity of FN.1 signature mutations

To understand the distribution, properties, and impact of signature mutation in S protein, we used COV2var (Feng et al. 2024), a SARS-CoV-2 mutation annotation database. Briefly, the COV2var integrates multiple tools including the Expasy ProtParam that was used for physico-chemical properties (Wilkins et al. 1999), GISAID for spatiotemporal distributions by lineage (Khare et al. 2021), the HyPhy software for both positive and negative selection (Kosakovsky Pond et al. 2020), I-Mutant 2.0 for protein stability (Capriotti et al. 2005), IUPred3 for disordered residues (Erdos et al. 2021), VaxiJen 2.0 for antigenicity of S mutations (Doytchinova and Flower 2007), IEDB Class I immunogenicity S mutations (Calis, 2013), and deep mutational scanning (DMS) approach for ACE2-binding affinity and antigenicity (Fowler and Fields 2014). Additionally, the Bloom Lab’s ACE2 binding calculator (Starr et al. 2022) and Bloom Lab’s antibody escape calculator (Greaney et al. 2022) were used to predict ACE2 affinity and the neutralizing antibody escape, respectively (Khare et al. 2021).

## Results

### Genotyping and patient characteristics in Botswana

**Figure 1.**
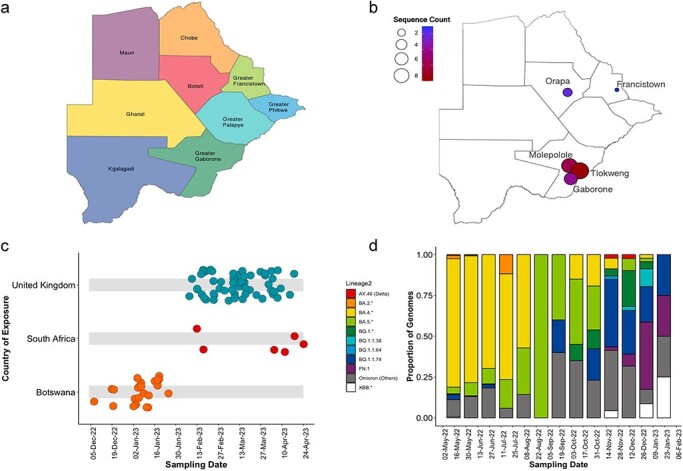
Distribution of SARS-CoV-2 FN.1, a sublineage of Omicron VOC. (a) Map of Botswana partitioned in nine COVID-19 zones, uniquely colored, (b) FN.1 sequence counts plotted on Botswana map partitioned into nine COVID-19 zones over December 2022 to February 2023. The frequency of FN.1 sequences and colored based on gradient. Botswana reported 22 sequences including Boteti (2), Greater Gaborone (*n* = 19), and Greater Francistown (1). (c) Changes in the genomic prevalence of Omicron lineage FN.1 overtime. The UK reported 59 sequences including England (*n* = 58) and Scotland (1). South Africa reported six sequences: including Mpumalanga (*n* = 1), Gauteng (*n* = 4), Free State (*n* = 1). (d) Proportion graph showing the landscape of SARS-CoV-2 lineages circulating between May 2022 and end of February 2023. This period correspondence to the emergence of BQ.1.1.64, BQ.1.1.74, and FN.1 lineages.

A total of 5370 complete genomes generated in Botswana by August 2023 were analyzed. Among these, 22 (0.41%) belonged to FN.1 (or BQ.1.1.74), clade 22E from the parental lineage BQ.1.1, a BA.5 sublineage of Omicron VOC. The samples of FN.1 were collected from five health facilities across three districts in Botswana between 7 December 2022 and 25 January 2023 (Fig. 1a–c). The median age of individuals from which FN.1 sequences were obtained was 42.5 years (Q_1_, Q_3_: 28.5, 49 years), with 13 (59.1%) being female (Table 1). When all the Omicron lineages BA.*, BQ.*, and FN.1 that were circulating in Botswana between May 2020 and January 2023 were analyzed by age and gender; a multi-regression analysis revealed a positive correlation of 1.85 (95% CI: 1.01–2.70) *P*-value <.01 for age, and 0.05 (0.01–0.06) *P* = .003 for gender (Supplementary Fig. S1). Among the individuals who had FN.1 with available clinical information shown in Table 1, 75% of cases exhibited common COVID-19 symptoms including cough and headache, and the median cycle threshold *(qCt)* values were 17.08 (Q1, Q3: 15.8, 28.5) for the N gene, and 21.77 (Q1, Q3: 20.18, 28.7) for the ORF 1a gene.

**Table 1.** Summary of the median age, gender distribution, vaccination status, and clinical features presented by participants at the time of sampling.

PID	Specimen date	Submission date	Age (years)	Sex	Origins	Location	Clinical presentation		CT values		Vaccination status	
							Symptomatic	Examination	N_gene_	ORF_gene_	Fully vaccinated	Vaccine
EPI_ISL_16521207	2023-01-03	2023-01-15	19	M	BWA	Gaborone	✓	Fever, cough, headache	ND	ND	✓	Pfizer
EPI_ISL_16521209	2023-01-04	2023-01-15	69	M	BWA	Gaborone	✓	Fever, stiff neck, shortness of breath	ND	ND	✓	AstraZeneca (Mordena^b^)
EPI_ISL_16554001	2022-12-07	2023-01-18	64	M	ZA	Tlokweng		ND	29.69	30.93	ND	
EPI_ISL_16554005	2022-12-19	2023-01-18	43	F	BWA	Molepolole	✓	Cough, headache	29.63	26.48	x	Johnson & Johnson
EPI_ISL_16554006	2022-12-20	2023-01-18	15	M	BWA	Molepolole	✓	Cough, headache, sore throat	17.06	16.62	✓	Pfizer
EPI_ISL_16554009	2023-01-09	2023-01-18	55	M	BWA	Gaborone	✓	Cough, sore throat, blocked nose	23.17	26.21	✓	Astrazeneca
EPI_ISL_16724221	2023-01-19	2023-01-30	35	F	BWA	Gaborone		ND	ND	ND	ND	
EPI_ISL_16724222	2023-01-06	2023-01-30	44	F	BWA	Molepolole		ND	ND	ND	ND	
EPI_ISL_16724223	2023-01-06	2023-01-30	43	F	BWA	Molepolole		ND	ND	ND	ND	
EPI_ISL_16724226	2023-01-12	2023-01-30	31	M	ZW	Tlokweng		ND	28.45	28.65	ND	
EPI_ISL_16724227	2023-01-16	2023-01-30	22	M	LES	Tlokweng		ND	ND	ND	ND	
EPI_ISL_16724228	2023-01-11	2023-01-30	83	F	BWA	Gaborone		^a^Asymptomatic, hypertension	17.08	21.77	✓	Sinovac
EPI_ISL_17191271	2022-12-20	2023-03-13	51	F	BWA	Gaborone	✓	Fever, cough, sore throat	12.63	15.14	ND	
EPI_ISL_17191272	2023-01-02	2023-03-13	47	M	BWA	Gaborone	✓	Fever, cough, chills	17.2	20.72	ND	
EPI_ISL_17191275	2023-01-18	2023-03-13	15	M	BWA	Orapa	✓	Cough, headache, shortness of breath	16.87	20.18	✓	Pfizer
EPI_ISL_17190207	2023-01-19	2023-03-13	24	F	BWA	Gaborone		ND	ND	ND	ND	
EPI_ISL_17190206	2023-01-18	2023-03-13	47	F	BWA	Francistown		ND	ND	ND	ND	
EPI_ISL_17190205	2023-01-05	2023-03-13	47	F	BWA	Molepolole		ND	ND	ND	ND	
EPI_ISL_17190219	2023-01-05	2023-03-13	26	M	BWA	Molepolole		ND	ND	ND	ND	
EPI_ISL_17190217	2023-01-07	2023-03-13	38	F	BWA	Gaborone		Asymptomatic	15.75	19.56	ND	
EPI_ISL_17190214	2023-01-12	2023-03-13	31	M	ZW	Tlokweng		^a^Asymptomatic	28.45	28.65	ND	
EPI_ISL_17190213	2023-01-25	2023-03-13	42	F	BWA	Orapa	✓	Cough, headache	18.4	22.53	✓	Johnson & Johnson Pfizer

a Port of entry (screening).

b booster dose.

ND: data not available.

BWA: Botswana; ZW: Zimbabwe; ZA: South Africa; LES: Lesotho.

N_gene_: SARS-CoV-2 nucleocapsid protein gene; ORF_gene_: SARS-CoV-2 open reading frame gene.

### Lineage classification and defining mutations

In addition to the 22 FN.1 sequences from Botswana (Fig. 1b), FN.1 sequences sampled between February 08 and 18 April 2023, were also identified in the UK (*n* = 59) and South Africa (*n* = 6) (Fig. 1c). FN.1 lineage did not exceed monthly prevalence over 1.33 × 10^−5^% throughout the pandemic (Q1, Q3: 6.40 × 10^−6^% to 2.64 × 10^−5^%) of globally sampled SARS-CoV-2 genomes between January 15 and 11 July 2023 (Supplementary Table S1). FN.1 likely emerged at the time when BQ.1-like lineages were mostly circulating in Botswana (Fig. 1d). The S protein’s mutational landscape indicates that BQ.1.1.74 is FN.1’s direct precursor. In addition to the 31 S protein mutations in BQ.1.1—S:T19I, S:A27S, S:G142D, S:V213G, S:G339D, S:R346T, S:S371F, S:S373P, S:S375F, S:T376A, S:D405N, S:R408S, S:K417N, S:N440K, S:K444T, S:L452R, S:N460K, S:S477N, S:E484A, S:F486V, S:Q498R, S:N501Y, S:Y505H, S:D614G, S:H655Y, S:N679K, S:P681H, S:N764K, S:D796Y, S:Q954H, S:N969–; BQ.1.1.74 features the signature missense variant K182E. FN.1 has the T478R mutation (DNA:1433C > G; position relative to WG:22 995) in the RBD (receptor-binding domain) and K182E (DNA:544A > G; pos:22106 G) in the NTD (N-terminal domain) (Fig. 2).

**Figure 2.**
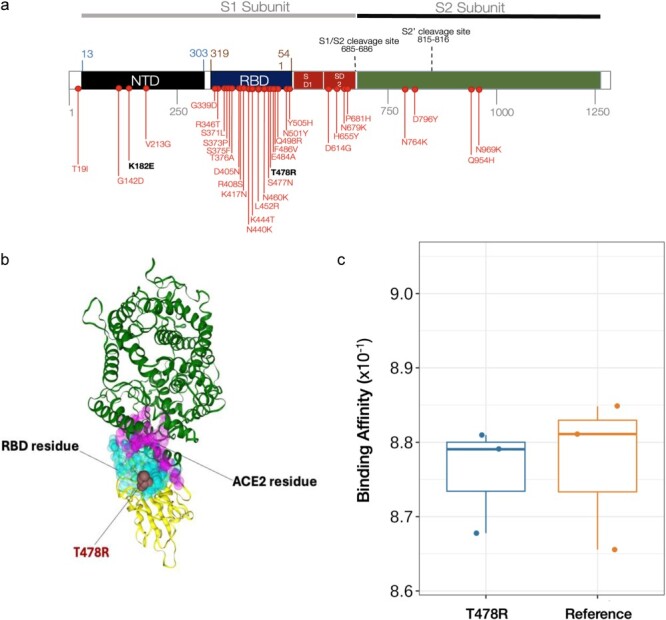
Mutation profiles and characteristics of FN.1. Spike protein changes in lineage BQ.1.1 and FN.1. The mutations common in BQ.1.1 relative to SARS-CoV-2 reference strain (NC_045512) encoded protein are represented in red. (a) The lineage defining mutations for FN.1 are indicated in black. The spike mutations in NTD S:K182E (nuc:22106 G), and RDB S:T478R (nuc:22995 G) for FN.1 are indicated in bold. The ACE2 affinity and immune escape scores of FN.1 relative to Omicron (BA.1) were 0.629 and 0.834, respectively. (b) Annotation of the S:T478R mutation on the 3D spike protein. (c) The boxplot comparing the binding affinities of spike proteins with T478R mutation and without (reference strain: NC_045512). (d) Bar plot showing the impact scores of key signature mutations of BQ.1.1.64, BQ.1.1.74, and FN.1 lineages. Properties assessed include antigenicity, pathogenicity, and immunogenicity. For these analyses, the VaxiJen tool was used to assess antigenicity, the IED tool for immunogenicity, and the MutPred2 software for pathogenicity. Based on the MutPred2 software, a score >0.5 indicates an increased likelihood of pathogenicity (Pejaver et al. 2020). An absolute change >0.0102 in antigenicity (three times the median across sites) is deemed significant, as is an absolute change exceeding 0.2754 for immunogenicity. In this context, antigens with a VaxiJen prediction score >0.4 are considered candidate antigens (Doytchinova and Flower 2007). An MHC I immunogenicity score >0 suggests a higher likelihood of eliciting an immune response.

**Figure 2.**
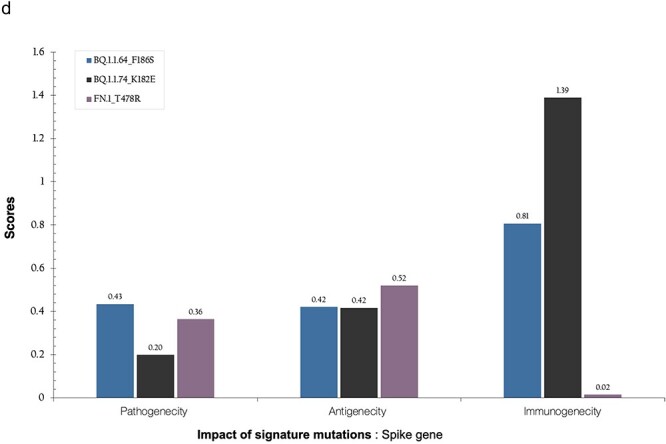
(continued).

### Impact of FN.1 signature mutations

The predicted pathogenicity score for T478R was 0.364, indicating a low likelihood of increased pathogenicity (Fig. 2b). Based on the MutPred2 software, a score > 0.5 indicates an increased likelihood of pathogenicity (Pejaver et al. 2020).

The mutation exhibited a slightly higher antigenicity score of 0.5201, compared to 0.5197 for the Wuhan reference sequence (NC_04415). Similarly, the immunogenicity score was 0.0153, in contrast to 0.00265 for the Wuhan reference. These shifts in antigenicity and immunogenicity have important implications for vaccine design and understanding immune responses. An absolute change >0.0102 in antigenicity (three times the median across sites) is deemed significant, as it is an absolute change exceeding 0.2754 for immunogenicity. In this context, antigens with a VaxiJen prediction score (Doytchinova and Flower 2007) >0.4 are considered candidate antigens. An MHC I immunogenicity score >0 suggests a higher likelihood of eliciting an immune response.

Based on the Bloom lab’s ACE2 binding calculator (Starr et al. 2022), a moderately positive impact on ACE2 affinity relative to a BA.2 baseline and expressed in log_10_ space. Thus, a score of +1 should be interpreted as 10× higher affinity. Also, we used the Bloom lab’s antibody escape calculator (Greaney et al. 2022) to predict the neutralizing antibody escape profiles of the FN.1 sequences relative to BA.2 which indicated that 42.2% of BA.2 induced neutralizing responses would act against FN.1. The comparison of the antigenicity, pathogenicity, and immunogenicity of key signature mutations of FN.1, BQ.1.74, and BQ.1.1.64 are shown in Fig. 2d.

### Landscape of circulating lineages in Botswana at the time FN.1 was identified

The empirical observations demonstrate that the SARS-CoV-2 lineage BQ.1.1 has undergone subsequent evolutionary diversification, yielding multiple discernible sublineages, independent of the pangolin designation. Using ML phylogenetic inference with branch supports tested using 1000 bootstrap replicates, we constructed a phylogenetic tree using 5370 sequences consisting of 99 lineages that circulated across the five previous COVID-19 epidemic waves in Botswana between March 2022 and August 2023. Using the sequences from Botswana, we examined the most probable ancestral clade of the most recent common ancestor of the sampled FN.1 sequences according to (i) mutational profiling and (ii) phylogenetic clustering. Despite the earliest sample of the 22 Botswana sequences being on 7 December 2022, based on the time scaled MCC tree of all the FN.1 sequences inferred by Bayesian analysis, FN.1 putatively emerged approximately on 22 October 2022 (95% HPD: 2 September 2022 to 24 November 2022); most probably in Botswana (posterior probabilities > .90) (Fig. 4a). Similar parameter estimates were shared by both coalescent prior runs such as tMRCA and inferred MCC tree topology (Supplementary Fig. S2).

**Figure 3.**
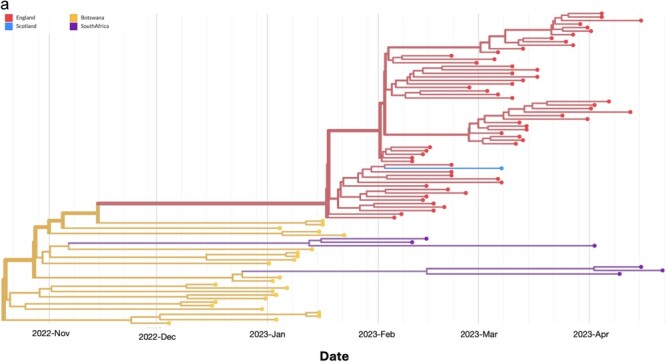
(a) MCC tree of 87 FN.1 global genomes including sequences generated in this study. These were generated in Botswana, South African, UK, and Scotland. The Discrete BSSVS SkyGrid model estimated the tRMCA on 22 October 2022 with Botswana at the origin seeding two introductions to South Africa and one to the UK. Both exports have caused onward transmission chains which is important for genomic surveillance. (b) Mapping inferred viral dissemination patterns of the SARS-CoV-2 Omicron sublineage FN.1 sequences from phylogeographic reconstructions based on discrete BSSVS SkyGrid model. Overall movement of the virus from Botswana to South Africa and UK shown.

**Figure 3.**
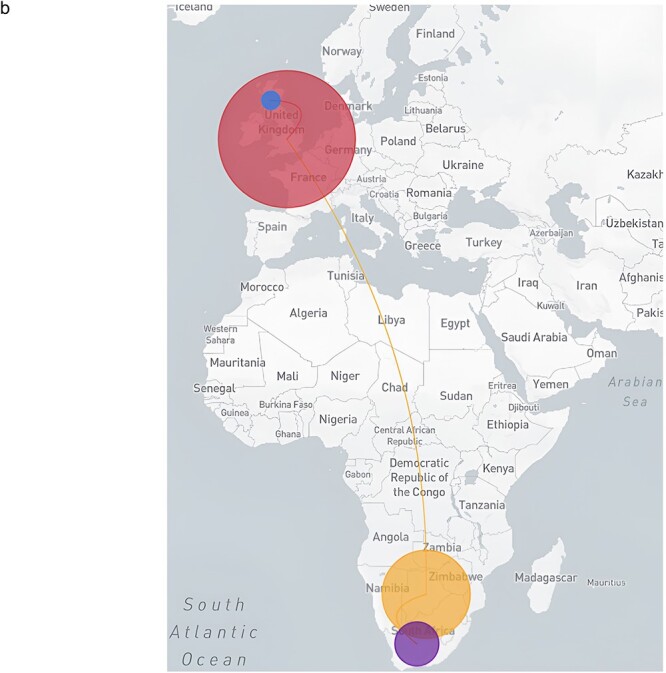
(continued).

When investigating all sequences that circulated in Botswana from May 2022 to February 2023, we observed a close clustering of FN.1 with BQ.1.1.* lineages (Supplementary Fig. S3). Two BQ.1.1.64 sequences were sampled on 9 July and 9 December 2022, 8 BQ.1.1.38 sequences were sampled between 26 December 2022, and 5 May 2023, and 62 BQ.1.1.74 sequences were sampled between 23 May 2022 and 24 January 2023. These BQ.1.1.74 sequences contained the closest relatives of FN.1, with the FN.1 cluster nested within the BQ.1.1.74 clade with >90% bootstrap support (Fig. 6a–c). The defining mutations of BQ.1.1.74 are ORF1b:N1191S and S:R346T, as compared to S:K182E, S:T478R, ORF1ab:N268S, and M:D3N for FN.1. Consistent with a Botswana origin of FN.1 is the fact that the earliest BQ.1.1.74 and FN.1 genome samples were from Botswana.

**Figure 4.**
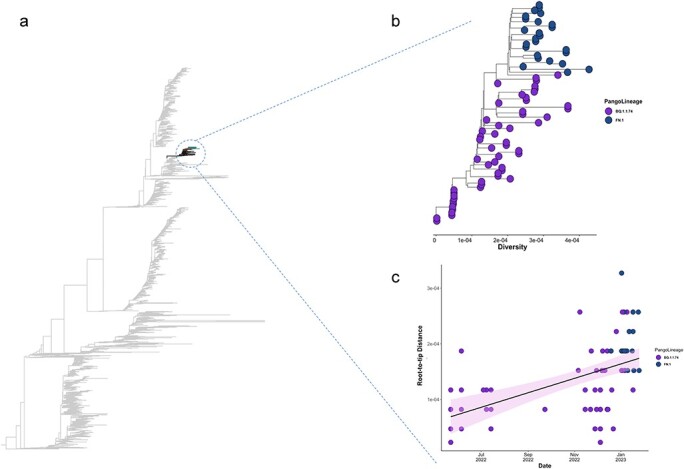
A ML tree of all the 5730 sequences from Botswana and root-to-tip plot showing SARS-CoV-2 lineage that is closely related to FN.1 lineage. (a) ML tree including all 99 SARS-CoV-2 lineages among the 5370 sequences from Botswana. The overall tree was rooted by the midpoint rooting. Among all the sequences, BQ.1.1.64, BQ.1.1.38, and BQ.1.1.74 were mostly on the basal of FN.1 (shown in Supplementary Fig. S2). Of these, **BQ.1.1.74** sequences represented the most significant statistical support for the FN.1 cluster (*P* > .90). (b) Expanded view of the FN.1 and BQ.1.1.74 sublineages extracted from the ML tree of the SARS-CoV-2 whole genome sequences characterised in Botswana. The diversity is represented on the *x*-axis of the branched tree. (c) The root-to-tip regression obtained from TempEst analysis for the sampled clusters of BQ.1.1.74 and FN.1 lineages, showing a relatively strong clock-like behaviour, the regression line (representing the estimated mean evolutionary rate) is shown with error buffers that represent 95% CIs.

### Spatio-temporal movement of FN.1

To understand the dispersion of FN.1 within Botswana and globally, we integrated sample locations and genomic sequence data within a phylodynamic analysis. As shown in Fig. 4b, the time-stamped phylogeny revealed that UK genomes (England, Scotland) were distinct from those sampled in Africa (South Africa, Botswana). This pattern suggests that the co-circulation of FN.1 SARS-CoV-2 lineages in Botswana primarily stemmed from local transmission rather than importation from an external source. Through ancestral location state reconstruction on the dated discrete phylogeny, Botswana emerged as a primary source of viral exportation of FN.1 to other countries (Supplementary Fig. S4).

### Evolutionary rate estimates, phylogenetic, and phylogeographic analysis

The molecular clock signal in these sequences was evaluated further using the 87 dated FN.1 sequences. Based on these 87 sequences, a regression of genetic divergence against sampling time confirmed that the FN.1 lineage is distinct from other Omicron lineages and that the dataset was suitable for molecular clock analysis (correlation coefficient = 0.67, *R*^2^ = 0.45). Under a strict clock model, the estimated mean evolutionary rate of the entire FN.1 lineage was 6.15 × 10^−4^ and 5.61 × 10^−4^ substitutions per site per year (s/s/y), with a 95% credibility interval ranging from 4.09 to 8.18 × 10^−4^ s/s/y and 3.66 to 7.66 × 10^−4^ s/s/y for the exponential and SkyGrid prior, respectively.

Phylodynamic analysis of full-length sequences from all genotypes of FN.1 (with credible intervals spanning from 1 September 2022 to 1 March 2023) using the SkyGrid model revealed that the overall effective population size exhibited exponential growth in early 2023, followed by a subsequent decrease (Supplementary Fig. S5). Furthermore, the population dynamics of the FN.1 lineage—inferred in the SkyGrid represented by the peaks—reflects the epidemiological data gathered for the FN.1 outbreak in the three countries between December 2022 and April 2023. Additionally, the peak of FN.1 cases occurred before 2023 (when most sequences were sampled), suggesting a substantial degree of under sampling of sequences in Botswana during this peak. The exponential effective population estimate is shown by the SkyGrid and estimate change over time as shown in the same plot.

## Discussion

We describe comprehensive assessment of detection, characterization, spread, evolution, and functional evaluation of the SARS-CoV-2 FN.1 lineage—a descendant of the Omicron BQ.1.1 lineage—that was initially detected in Botswana among the 22 cases collected from five health facilities across three districts in Botswana between 7 December 2022, and 25 January 2023. Ongoing surveillance efforts in Botswana following the first Omicron lineage discovery facilitated the tracking, diversification, and subsequent emergence of BQ.1* lineages. During the period when we sequenced FN.1 and two other BQ lineages—BQ.1.1.64 and BQ.1.1.74—, there was no discernible increase in either recorded COVID-19 cases or deaths. Based on the sampling dates and phylogenetic analyses, FN.1 likely emerged and circulated in Botswana and was exported globally to the UK and South Africa whereby this sublineage was also identified. Apart from FN.1, the BQ.1 lineage has evolved yielding multiple sublineages circulating globally. Additionally, BQ.1 sublineages that were reported in Botswana included BQ.1.1, BQ.1.1.38, BQ.1.1.64, and BQ.1.1.74. Among these, three were first sequenced and reported in Botswana including BQ.1.1.64 (7 September 2022), BQ.1.1.74 (23 May 2022), and FN.1 (7 December 2022). It is noteworthy that both BQ.1 and BQ.1.1 were reported in Nigeria (July 2022) (Akash et al. 2023).

Convergent evolution of SARS-CoV-2 Omicron subvariants led to the emergence of the BQ.1.1 variant, a precursor to BQ.1.1.74 (Ito et al. 2023). Phylogenetically, FN.1 is likely derived from BQ.1.1.74 which is also more prevalent in Botswana (Supplementary Fig. S5). FN.1 has two lineage defining mutations in the S protein; S:K182E and RBD S:T478R of which the BQ.1.1.74 does not have S:K182E. The BQ.1 lineage and its sublineages harbour key mutations (S:R346T, S:K444T, S:L452R, S:N460K, or S:F486V) which potentially have functional importance (Groenheit et al. 2023, Ito et al. 2023). Specifically, these two mutations have been associated with enhanced binding affinity of the S protein to hACE2 and are likely to facilitate evasion of antiviral humoral immunity induced by both vaccination and natural SARS-CoV-2 infections (McCarthy et al. 2021, Zhang et al. 2022). It is likely that the fitness benefits of these two mutations enabled BQ.1 and its sublineages to spread more efficiently than the proceeding lineages (Tamura et al. 2023).

Unlike BA.5 and most of its sublineages (including BQ.1) that have S-gene target failure, (SGTF) mainly characterized by the Spike DH69/V70 deletion (McCarthy et al. 2021, Lythgoe et al. 2023). FN.1 does not have this 69/70 spike deletion (SGTF). The S:K182E and S:R346T mutations reported are rare, and each has a global prevalence <0.01%. Nevertheless, R346T is a known monoclonal antibody resistance mutation (Sheward et al. 2022, Qu et al. 2023). Additionally, the S:K478R mutation in combination with F486 has recently been identified in XBB.1.16 and other BQ* sublineages (Faraone et al. 2023). S:K478R was also observed in FN.1 sequences from South Africa and the UK. Given the inferred rapid growth rate of the FN.1 lineage at the end of 2022 (Chen et al. 2022), the S:K478R mutation that it carried may have conferred a significant advantage to this lineage over its parent lineage (BQ.1.1): with FN.1 even potentially exhibiting a transiently faster growth rate than XBB.1.5 (Chen et al. 2022). This is likely the lineage that went on to dominate global SARS-CoV-2 populations during the first nine months of 2023. Similar to XBB.1.5, FN.1 is likely representing a lineage with some capacity to evade immunity elicited by BA.4/5 (Huo et al. 2023, Ma et al. 2023). The most plausible reason it did not disseminate more widely is simply that there were simultaneously many other lineages with similar immune evasion phenotypes when it emerged at the end of 2022.

We investigated a lineage that was closely related to FN.1 using phylogenetic analysis including all 5370 sequences generated in Botswana by 1 October 2023. Our focus was on the clustering of FN.1 with other BQ.1 lineages. The 5370 sequences were classified into 99 lineages (Supplementary Table S2) among which the BQ.1.1.74 cluster of genomes was basal to the FN.1 cluster (Fig. 6). The 64 cases of lineage BQ.1.1.74 were sampled between 23 May 2022 and 24 January 2023. Given (i) that most BQ.1.1.74 sequences were collected between 1 December and 16 December 2022, and the first case of FN.1 was reported on 7 December 2022, from an elderly male (64 years) in Tlokweng (community under Greater Gaborone COVID-19 zone), (ii) that both lineages were closely related phylogenetically, and (iii) that they were identified from the same COVID-19 zone of Botswana, we speculate with caution the possibility of that the FN.1 sublineage evolved from a BQ.1.1.74 progenitor in Botswana.

However, FN.1 and its likely BQ.1.1* progenitors cluster distinctly from other BA.* lineages, and XBB, both of which were circulating in Botswana during the period when it is assumed that FN.1 emerged (late-2022 to early 2023). The earliest confirmed sample of FN.1 was isolated in Greater Gaborone, Botswana on 7 December 2023, and was submitted to GISAID on 18 January 2023 (Table 1). The FN.1 tMRCA of October 2022 corresponded to a slightly earlier time-period of the first case reported in Botswana (Fig. 4). The mean evolutionary rate of the FN.1 lineage [6.15 × 10^−4^ (4.09 to 8.18 × 10^−4^ s/s/y)] was lower than those reported for some other SARS-CoV-2 lineages but was still well within the estimated 95% CIs for these other lineages [7.3 × 10^−4^ (5.95 × 10^−4^–8.68 × 10^−4^ s/s/y] (Bukin et al. 2021). This translates to a slower but not significantly different evolution of the FN.1 lineage over time. Although the full public health impact of this variant is unknown, the available genomic and epidemiological data suggests that this variant had at best, a minor selective advantage in Botswana over contemporary lineages (based primarily on low case counts). Similar to other variants, we hypothesize that FN.1 may have emerged through intra-host evolution and spread via contact. The mutation S:K182E is one of the defining mutations of BQ.1.1, FN.1, and BQ.1.1.74, all of which (i) share similar mutation profiles, (ii) were initially identified in Botswana, and (iii) circulated there at low frequencies. The sequences obtained from the three countries where FN.1 was sampled likely reflect the extensive sequencing capabilities of these countries rather than significant prevalence of the variant. Resembling other lineages with immune evasion properties, this lineage competed well for acquiring new hosts, but it did not exhibit potential to spread facing a robust immune landscape of Botswana that had previously been achieved through extensive vaccination efforts and high incidence of natural Omicron infections (Carabelli et al. 2023).

Our study has some notable limitations. Some of our inferences rely on the accuracy of sampling location and dating records in the GISAID database. In late 2022, rates of testing regionally and globally were influx with a steadily declining trend at the time when FN.1 emerged, and hence there were likely many FN.1 infections left unsampled in Botswana and neighbouring countries; under-sampling may have therefore biased inference of the possible original location of FN.1 to Botswana or South Africa (the southern African countries with the strongest ongoing surveillance) (Tegally et al. 2022b). Also, we cannot definitively determine if the relatively low numbers of reported cases during the time-period when FN.1 was circulating accurately reflected low levels of FN.1 transmission or was simply a consequence of declining COVID-19 testing rates. We accordingly acknowledge that our preliminary estimate of evolutionary rate of FN.1 may be relatively less accurate than if more intensive sampling had yielded more examples of this lineage. Nevertheless, the global temporal pattern, particularly observed in countries with extensive sequencing efforts, such as the UK, suggests that only low proportion of FN.1 cases circulated, and the Botswana FN.1 sequences were basal progenitors of all the sequences (Supplementary Fig. S6). We therefore postulate that among contemporary SARS-CoV-2 variants with increased transmission potential and immune evasiveness, FN.1 may have simply been a good contender that ultimately failed to accumulate as many fitness enhancing mutations as those lineages that ultimately came to dominate SARS-CoV-2 populations during 2023 (Fig. 2d). It is nevertheless of great value to explore the functional implications of both Spike and non-Spike protein modifications in “almost important” lineages such as FN.1: especially when such lineages arise in parts of the world where globally dominating variants such as Omicron first emerged and have been circulating the longest. While the functional attributes of the mutations characterizing the FN.1 lineage remain unexplored, our findings emphasize the critical importance of well-coordinated molecular surveillance systems at both national and global levels. Such systems play a pivotal role in the timely identification and characterization of emerging lineages, thereby guiding the global responses to the COVID-19 pandemic and its aftermath.

In conclusion, we have established a response system to continuously monitor and investigate emerging SARS-CoV-2 lineages. This study describes a domestic SARS-CoV-2 lineage, FN.1, and performs downstream analyses as part of SARS-CoV-2 genomic surveillance in Botswana. This approach is crucial for assessing the potential for another Omicron-like outbreak.

## Supplementary Material

veae095_Supp

## Data Availability

The data underlying this article are available in the article and in its online Supplementary Material. The SARS-CoV-2 genome sequences in this study have been deposited in GISAID and the accession numbers are shown in Table 1.
