# Supplementary material for: Emergence of Omicron FN.1 a descendent of BQ.1.1 in Botswana
Source: Virus Evol. 2024 Nov 22;10(1):veae095. doi: 10.1093/ve/veae095 (PMC11666700; doi:10.1093/ve/veae095)
Supplement: veae095_Supp [file veae095_supp.zip › New folder/Supplementary_material-20240626-finalcheck-0827.docx]

**Supplementary material**


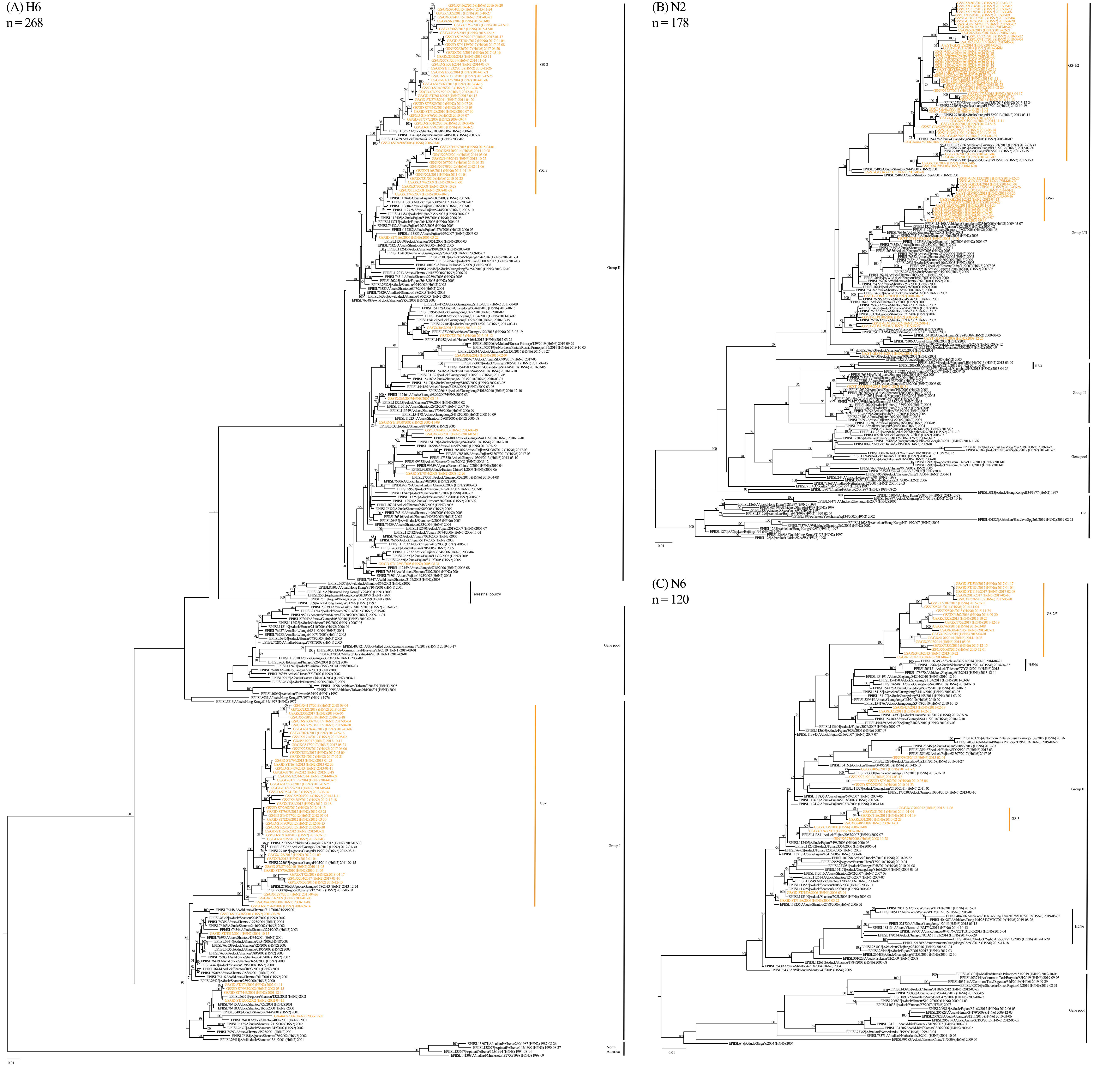


**Figure S1.** Maximum likelihood phylogenies of the surface protein genes. (A) H6, n=268; (B) N2, n=178; (C) N6, n=120. The viruses isolated in this study are colored orange (abbreviated names). Viruses isolated from minor poultry clustered in the clade of terrestrial poultry. Branch scale represents 0.01 substitutions per site. Bootstrap values from 1,000 pseudo-replicates are shown on selected branches. Abbreviations for host species: GS (Goose). Geographical abbreviations: GD (Guangdong), ST (Shantou, Guangdong), GX (Guangxi). Readers can zoom in to view the strain names with clarity.





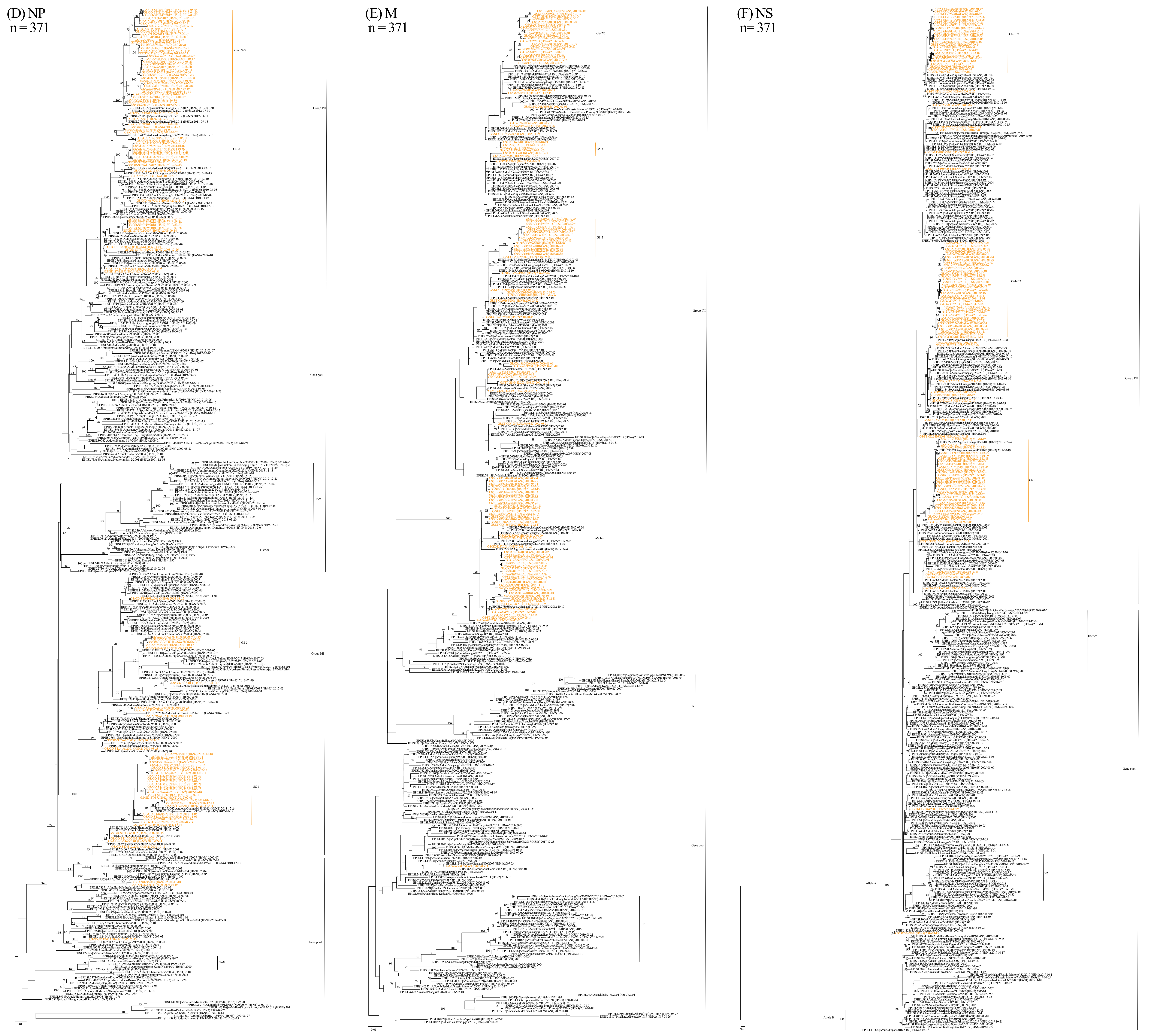


**Figure S2.** Maximum likelihood phylogenies of the internal genes. (A) PB2, n=371; (B) PB1, n=371; (C) PA, n=371; (D) NP, n=371; (E) M, n=371; (F) NS, n=371. Annotation is the same as that defined in the legend of Figure S1. Alleles A and B are indicated with brackets in the NS gene tree.

**­­
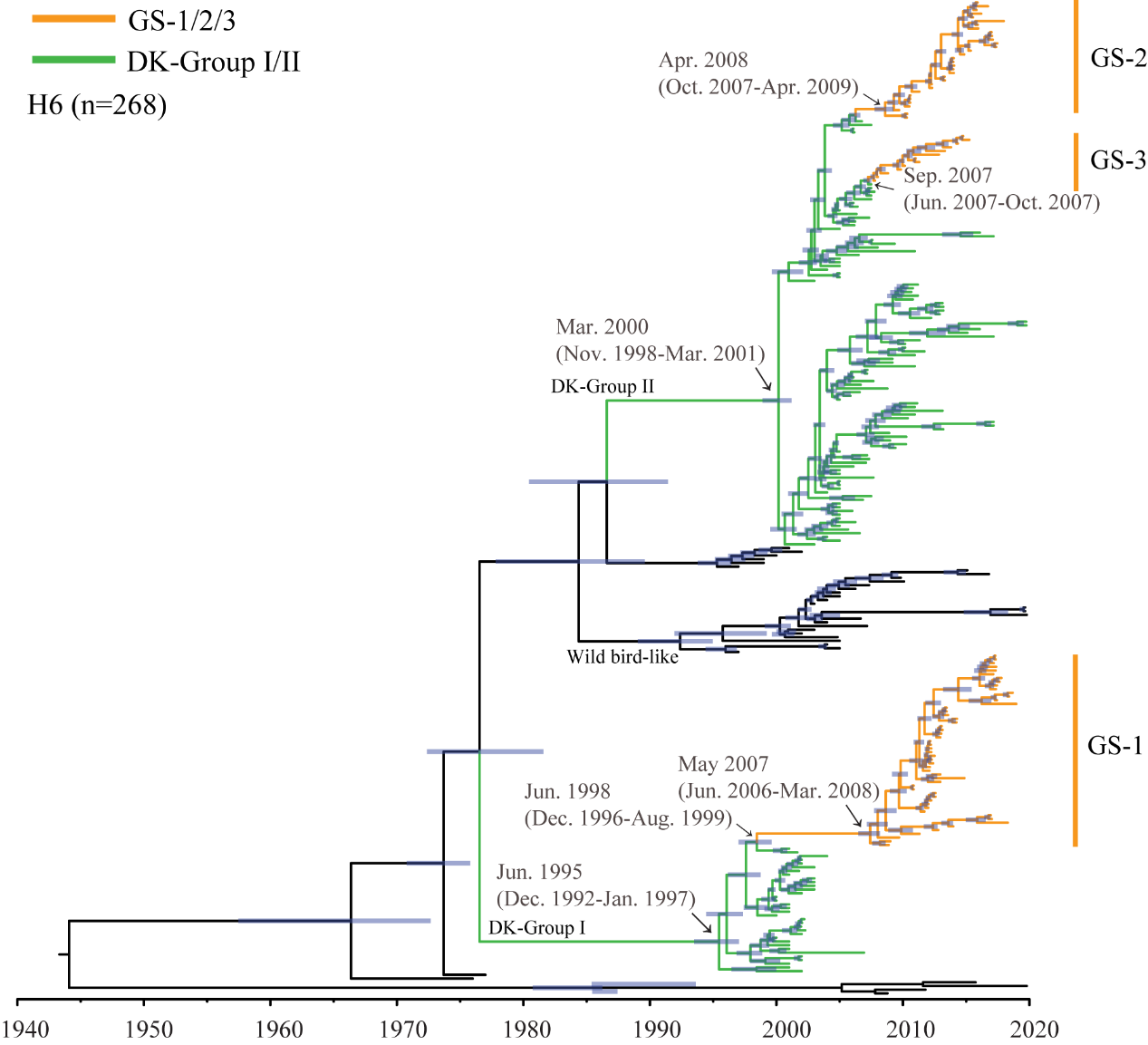
**

**Figure S3.** Maximum clade credibility tree of the H6 HA (n=268). H6 clades established in ducks and geese are colored in the phylogenetic tree. The most recent common ancestors of these clades are marked on the nodes shown by the arrows. The 95% highest posterior density intervals (95% HPD) of the TMRCA estimates are indicated in parentheses.

**
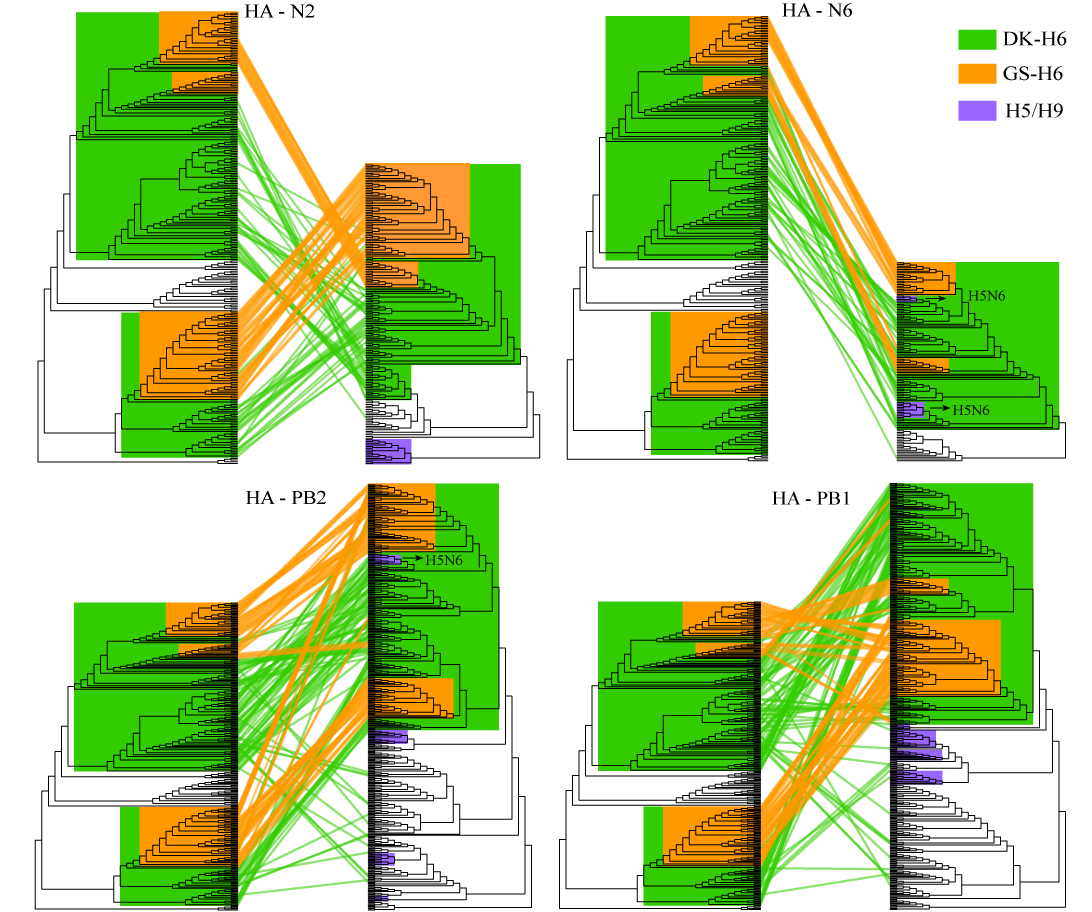

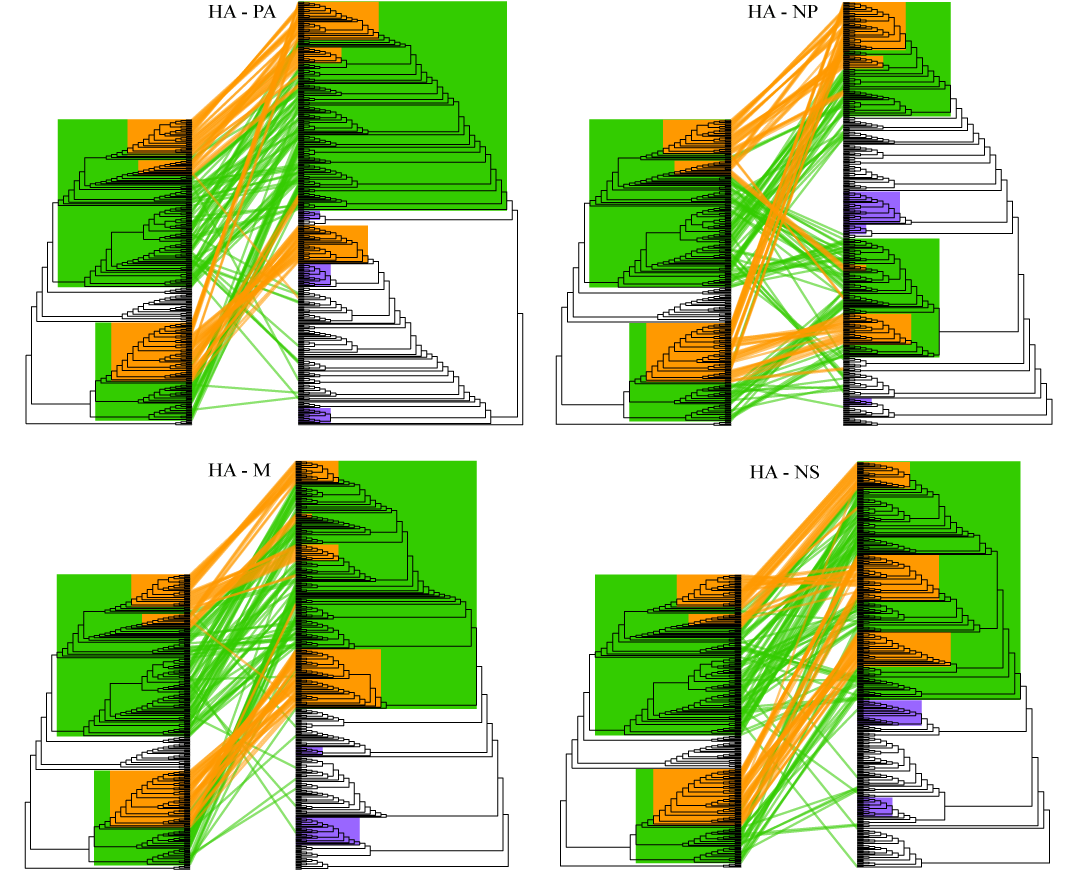
**

**Figure S4.** Reassortment patterns of H6 viruses established in ducks and geese. Tanglegrams were built based on maximum likelihood (ML) phylogenies with unscaled branch lengths, with the ML HA tree on the left and the mirrored tree of the other gene on the right. Evolutionary positions of the duck (green) and goose (orange) H6 viruses are linked by lines in each paired phylogeny. H5 and H9 viruses established by a purple blue background. Viruses from the gene pool (wild bird-like) are not colored in the gene phylogenies. Gene exchange between H5 and H6 viruses was highlighted by an arrow. Abbreviations: DK, duck; GS, goose.

**Table S1.** Marginal likelihood estimates for each model combination for the BEAST analysis.

| Clock^a^ | Tree prior | Marginal likelihood value |
| --- | --- | --- |
| UCLD | Bayesian SkyGrid coalescent | -29,295 |
| UCLD | GMRF Bayesian Skyride | -29,331 |
| UCED | Bayesian SkyGrid coalescent | -29,349 |
| UCED | GMRF Bayesian Skyride | -29,384 |

^a^ UCLD, uncorrelated relaxed clock with a log-normal distribution; UCED, uncorrelated relaxed clock with exponential distribution.

**Table S2.** Parameter estimates of variable ω values among branches under branch model in CodeML analysis and a likelihood ratio (LRT) test comparing.

| Model^a^ | ω_Wild bird-like_ | ω_DK-Group I_ | ω_DK-Group II_ | ω_GS-1_ | ω_GS-2_ | ω_GS-3_ | lnL^b^ | LRT |
| --- | --- | --- | --- | --- | --- | --- | --- | --- |
| H_0_ | 0.14 | 0.14 | 0.14 | 0.14 | 0.14 | 0.14 | -28392.58 | / |
| H_branch_ | / | 0.13 | 0.13 | 0.20 | 0.38 | 0.92 | -28380.25 | p<0.001 |
| H_clade_ | 0.09 | 0.10 | 0.16 | 0.18 | 0.15 | 0.16 | -28336.48 | p<0.0001 |

^a^ The topology, specific branches or clades used for estimates of ω_branch_ and ω_clade_, are presented in Figure S3.

H_0_: ω_Wild bird-like_=ω_DK-Group I_=ω_DK-Group II_=ω_GS-1_=ω_GS-2_=ω_GS-3,_

H_branch_: ω_DK-Group I_=ω_DK-Group II_≠ω_GS-1_≠ω_GS-2_≠ω_GS-3_

H_clade_: ω_Wild bird-like_≠ω_DK-Group I_≠ω_DK-Group II_≠ω_GS-1_≠ω_GS-2_≠ω_GS-3_

^b^ lnL, Log-likelihood Values

/, no test

**Table S3.** Amino acid substitutions at host-jump nodes.

| Node^a^ | Nonsynonymous mutation sites^b^ (H3 numbering) |
| --- | --- |
| Wild bird-like | A128T |
| DK-Group I | K56R, T136S, S143T, K165R*, S208R, K259R*, S263R*, K452R*,  D 487N*, D493G, E501T*, M532L*, L536V |
| GS-1 | V54I, G63D, T65N, S136T, V163I, K173R, D187N, E190A, N210S, A212S, A219E, A222T, K238E, L300H, R367K, I374V, N446I, G493S, V536L |
| DK-Group II | Q25K*, G63E*, I66L*, T92N*, Q93R*, S133N*, N137S, S138R*, T142G*, T156P*, A159S*, Y168F*, N169R*, N173D*, Q174K*, P175S*, I176V*, P186T, N189T*, T193I*, D199N, K213R*, Y233F*, V236I*, K259R*, N262D*, V267I*, A285E*, L288I, K372R*, D396G*, K450R*, I478N*, K496F*, R499K*, L510F* |
| GS-2 | E63D, V105A, R117G, S137N, E190Q, N199D, A218M, Q226L, N271D, I284V |
| GS-3 | R117K, S140R, T186A, E190Q, I288V |

^a^ Nodes of cross-species transmissions indicated by the arrows in Figure S3.

^b^ Positions located within the 130-loop, 190-helix and 220-loop of the receptor binding domain are highlighted in grey. Residues estimated to be positively selected by MEME (p≤0.05) are highlighted with red letters. Asterisks indicate that mutations at these sites in ducks are maintained in geese after cross-species transmission.

**Table S4.** Amino acid deletion patterns of the N2 NA genes of H6 viruses isolated in geese.

| Year | No. of viruses with the indicated deletion pattern | | | No. of sequences  (No. of deletions) |
| --- | --- | --- | --- | --- |
|  | Δ66-75 | Δ63-81 | Δ69-78 |  |
| 2000-2005 |  | 1 | 1 | 8(2) |
| 2006-2010 | 1 |  | 5 | 12(6) |
| 2011-2018 |  |  | 10 | 51(10) |
| Total | 1 | 1 | 16 | 71(18) |
